# Combining X-rays, neutrons and electrons, and NMR, for precision and accuracy in structure–function studies

**DOI:** 10.1107/S205327332100317X

**Published:** 2021-05-04

**Authors:** John R. Helliwell

**Affiliations:** a Department of Chemistry, University of Manchester, Manchester, M13 9PL, United Kingdom

**Keywords:** X-rays, neutrons, electrons, NMR, structure and function

## Abstract

The distinctive features of the physics-based probes used in understanding the structure of matter focusing on biological sciences, but not exclusively, are described in the modern context. This is set in a wider scope of holistic biology and the scepticism about ‘reductionism’, what is called the ‘molecular level’, and how to respond constructively. These topics will be set alongside the principles of accuracy and precision, and their boundaries. The combination of probes and their application together is the usual way of realizing accuracy. The distinction between precision and accuracy can be blurred by the predictive force of a precise structure, thereby lending confidence in its potential accuracy. These descriptions will be applied to the comparison of cryo and room-temperature protein crystal structures as well as the solid state of a crystal and the same molecules studied by small-angle X-ray scattering in solution and by electron microscopy on a sample grid. Examples will include: time-resolved X-ray Laue crystallography of an enzyme Michaelis complex formed directly in a crystal equivalent to *in vivo*; a new iodo­platin for radiation therapy predicted from studies of platin crystal structures; and the field of colouration of carotenoids, as an effective assay of function, *i.e.* their colouration, when unbound and bound to a protein. The complementarity of probes, as well as their combinatory use, is then at the foundation of real (biologically relevant), probe-artefacts-free, structure–function studies. The foundations of our methodologies are being transformed by colossal improvements in technologies of X-ray and neutron sources and their beamline instruments, as well as improved electron microscopes and NMR spectrometers. The success of protein structure prediction from gene sequence recently reported by CASP14 also opens new doors to change and extend the foundations of the structural sciences.

## Abstract 2

The distinctive features of the probes used in understanding the structure of matter focusing on biological sciences, but not exclusively, are described in the modern context to minimize the consequences of artefactual information in data interpretation. The precision and accuracy of both data and technique are revisited. A variety of structural results are described that reach beyond reductionism to the whole biological organism. All these aspects open new doors to change and extend the foundations of the structural sciences.

## 1. Introduction   

W. Lawrence Bragg (1968 ▸) wrote on X-ray crystallography that: ‘I have often been asked ‘Why are you always showing and talking about models? Other kinds of scientists do not do this’.’ Bragg answered that ‘The investigator seeks a structural plan, a map that shows all the atoms in their relative positions in space. No other branch of science is so completely geographical; a list of spatial coordinates is all that is needed to tell the world what has been discovered’.

**Figure 1:**
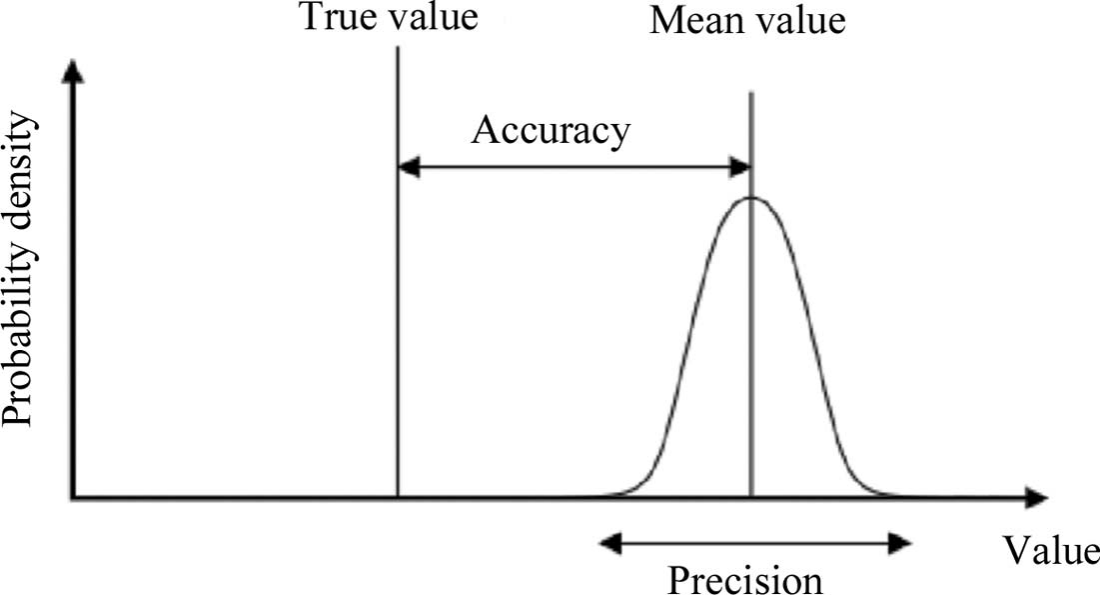
The concepts of precision and accuracy in measurement science.

So, we can see atoms with crystallography. We now must also ask: when are the atoms that we see in our electron density, nuclear density or electrostatic potential maps in the correct positions to explain a function? We need either an assay of function or a complementary technique providing a consensus or a predictive force from our crystal structure (*e.g.* site-directed mutagenesis for a protein where we can change the function such as the rate of an enzyme reaction based on its structure). These considerations drive to the core words in science of precision and accuracy (Fig. 1 ▸). When there is a correct match between one method’s measurements and a model interpretation, this equals precision. When a second method is used its measurements lead to a refined model, which can also be precise in the same way. But when both methods agree then we reach accuracy. There are other common words in measurement science to be mentioned. These are systematic error and random error. With any one method both types of error occur. Systematic errors one strives to remove completely if possible. So, in the use for example of an electronic area detector, it must be calibrated for dealing with its systematic spatial distortions or systematic non-linearity of response. These calibrations may also change with time, such as weeks to months, and recalibration to avoid systematic errors is needed. Another core point was made by the IUCr Working Party on ‘Expression of Uncertainty in Measurement’, and on types of error (Schwarzenbach *et al.*, 1995 ▸): ‘a measurement result is considered complete only when accompanied by a quantitative statement of its uncertainty’. I discuss this more in Section 4.2.

These terms have been considered from the charge density point of view by Sanjuan-Szklarz *et al.* (2020 ▸), the central issue being the use, in general, of spherical electron densities rather than the physically more reasonable aspherical electron densities. As their Fig. 6 nicely shows, the truth is not known and so accuracy could, even should, be replaced by a reference structure, and the neutron crystal structure is their reference. There is a commonality of that approach with the one I describe here for protein crystallography, but since it is at a lower, *i.e.* less good, diffraction resolution the reasons to make the neutron protein crystal structure the reference instead are: the structural completeness with experimentally determined hydrogens for the ionizable amino acids, the use of physiological relevant temperature and the absence of X-ray or electron radiation damage. That said, as Joachim Frank emphasized (see below), the crystal itself is not the ‘native state’.

It is important, indeed vital, to retain a big picture. So, in biology we need the hierarchies of organization and thereby of length scales of molecules, macromolecules, viruses and organelles into the complete organism, *e.g.* an animal, and even further, into whole populations and ecosystems. In similar vein we seek to understand the structural dynamics from femtosecond to second. Structural dynamics can include conformational change, atomic vibration or bond making and breaking. To this end we isolate molecules or complexes which in size reach a limit at the several hundred Å scale with *e.g.* a virus particle. The method of electron tomography can be used to view molecules *in situ*, such as the pioneering study of coronavirus particles (Almeida & Tyrrell, 1967 ▸).

Crystallography’s landscape of determining structures now coexists with DeepMind’s prediction of a protein 3D structure from a gene sequence as seen in CASP14, Critical Assessment of Structure Prediction of Proteins [for a short resume see Helliwell (2020 ▸)]. At CCP4 2021 McCoy reported that all models were good for molecular replacement (McCoy, 2021 ▸). A key question for the field of protein crystallography is: will DeepMind share its protein fold prediction software or provide a webserver for uploading gene sequences?

Flexible proteins and complexes are resistant to crystallization and electron cryo-microscopy (cryoEM) has undergone great strides in resolution capability in recent years [see Kühlbrandt (2014 ▸) for an overview]. For cryoEM, freezing a single molecular complex is quite possibly more favourable than freezing a >1 µm protein crystal given the possibility of cryo-artefacts. In its results cryoEM shows quite a range of resolutions across a structure, but so does crystallography with *B* factors, some very large to the point that such a part of a structure shows no significant density.

These considerations bring me to a more detailed scrutiny of our probes of the structure of matter. Vainshtein’s (1964 ▸) book provides an excellent survey of our diffraction probes: X-rays, electrons and neutrons. In particular, neutrons give the nuclear positions, X-rays the atomic electron density and electrons the atomic electrostatic potential. Such an overview has to my knowledge not been attempted since, which led me to consider writing this article.

## 2. Pairwise comparisons of probes   

### 2.1. Element identification   

The atomic scattering factor of atoms for X-rays steadily increases for increasing atomic number. The resonance effects (‘anomalous dispersion’) provided via the X-ray wavelength tuning to and around an individual element *K* or *L* absorption edge give X-ray diffraction an exquisite sensitivity to identifying a metal atom, especially bimetallic cases of neighbouring atomic number. The example with the most X-ray wavelengths, 11, to investigate a zinc-substituted gallium phosphate involving partial occupancy is that of Helliwell *et al.* (2010 ▸). By tuning to each of the zinc and gallium *K* absorption edges a very precise crystal structure was determined.

**Figure 2:**
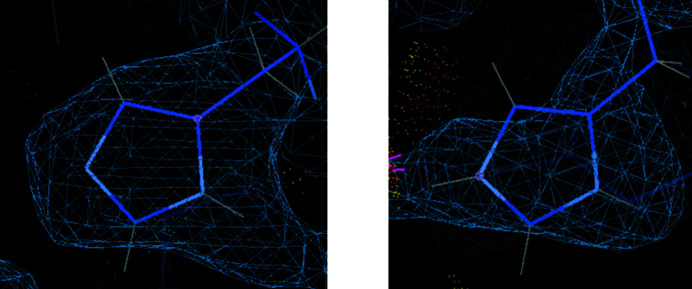
The exquisite sensitivity of neutrons as a probe for determining the protonation states of histidine, a frequent player in enzyme mechanisms. Left, His24 singly protonated; right, His180 doubly protonated histidine in concanavalin A (PDB code 2yz4; Ahmed *et al.*, 2007 ▸). This figure was made with *Coot* (Emsley & Cowtan, 2004 ▸).

The nuclear scattering for different isotopes using neutrons also provides an exquisite sensitivity with contrast variation involving for example hydrogen and deuterium. Likewise, there are element-to-element variations but these are much less marked than when using X-rays. A particularly attractive feature of the neutron scattering factors is that deuterium is as good a scatterer for neutrons as carbon. This is exploited in neutron macromolecular crystallography to determine the protonation states of ionizable amino-acid residues (aspartic acid, glutamic acid, histidine and lysine). Fig. 2 ▸ shows the nuclear density map for a single and doubly protonated histidine (Ahmed *et al.*, 2007 ▸).

Electrons also show a steadily increasing variation of the electrostatic potential with atomic number. They are much more sensitive to hydrogens than X-rays. For a biological molecule known from chemical analysis to contain an electron-dense metal centre, the location of those metal atoms can be found. However, to my knowledge, it is untested whether electrons can be used to determine bimetallic cases of closely similar atomic number.

Both electrons and X-rays will very likely change the oxidation state of a metal. With X-rays there is the important exception however of a femtosecond-time-range pulsed X-ray laser where the ‘diffract before destroy’ approach (Neutze *et al.*, 2000 ▸) is applicable.

### 2.2. Sample scattering power   

Perhaps surprisingly this was not introduced as an equation until the work of Andrews *et al.* (1988 ▸) – see their Table 3 comparing microcrystal diffraction of both chemical and protein microcrystallography. A different approach was adopted by Henderson (1995 ▸), namely that for naturally occurring biological material, electrons at present provide the most information for a given amount of radiation damage. The study involved comparing X-rays, neutrons and electrons. This result concurs with the Vainshtein (1964 ▸) approach of comparing the three probes and where electron scattering is the strongest, so the very smallest crystals can be used. The obvious utility of the Henderson (1995 ▸) analysis encouraged the development of cryoEM for single particles of biological macromolecules. Once a crystal is grown, or should I say growable to above a size of ∼0.1–1 µm, then the issue changes. Namely, that electrons are overtaken by X-rays in their utility for structure analysis. Furthermore, if the crystal grows to ∼1 mm^3^ or more then neutrons become viable and are by far the best because they yield a complete structure with hydrogens, at physiological temperature and free of radiation damage. Table 1 ▸ compares the fundamentals of X-rays and electrons, listing their pros and cons. Since neutrons are the ideal probe for crystallography, their use is purely one of practical challenges and these are compared with X-rays and electrons in Table 2 ▸. There is one fundamental aspect of neutron crystallography which is that analysis of a neutron protein crystal structure requires an X-ray crystal structure.

### 2.3. The important role of NMR   

NMR can determine protein structures in solution, without crystallization, and so is the only way forward for *e.g.* an intrinsically disordered protein. But the role of NMR has three additional advantages when combined with a crystal structure. Firstly, the NMR structure is actually an ensemble-of-structures fit to the measurements. This means that where the protein polypeptide chain is particularly flexible, and the X-ray crystal structure electron density has disappeared, the NMR ensemble fit still shows the range of positions for that portion. Secondly, the relatively static core of a protein as seen by the X-ray crystallography in the NMR data still shows dynamics. This was highlighted by Wüthrich & Wagner (1975 ▸) with respect to aromatic side chains such as phenylalanine where distinctly different NMR spectra could be seen showing three possibilities: spinning, 180° flip or static. Thirdly, the application of NMR to a crystal allows for both the ordered and disordered structures in the sample to be studied simultaneously. The book *NMR Crystallography* (Harris *et al.*, 2009 ▸) surveys a large number of examples of this; ch. 27 by Middleton (2009 ▸) is devoted to structural biology applications. Fig. 27.2 of Middleton shows the effects of sample preparation in solid-state NMR, comparing polycrystalline and nanocrystalline, which are ‘practically identical’, and thirdly, lyophilized ubiquitin protein which shows poor resolution indicating structural heterogeneity. Middleton (2009 ▸) also points out that where a crystal is available but does not diffract, or shows limited diffraction, then NMR crystallography could be applied to study protein–ligand binding on soaking with ligand. Lewandowski *et al.* (2015 ▸) used solid-state NMR to investigate protein and solvent motions of nano- and microcrystalline, fully hydrated, protein GB1 at temperatures from 105 to 280 K. GB1 is ‘a small globular protein specifically binding to antibodies’. Their study describes a hierarchical change in dynamic behaviour (see especially their Fig. 4) showing ‘a unified description of the essential conformational energy surface, relating the amplitude and activation of solvent, sidechain, and backbone motions in a hierarchical distribution, as well as unambiguous identification of NMR line broadening at cryogenic temperatures’. Stepping outside protein examples, Bryce (2017 ▸) provides a topical review of the use of NMR crystallography across a wide range of materials spanning inorganics and organics as well as a protein. The IUCr established a Commission on NMR Crystallography and Related Methods in 2014 in recognition of its importance (https://www.iucr.org/iucr/commissions/nmr-crystallography).

### 2.4. Diverse further frontiers in pursuit of structural accuracy   

There are diverse further important frontiers in the analytical characterization of 3D structures. These include mass spectrometry, X-ray absorption spectroscopy and infrared spectroscopy.

It is important to recall that, such as during my doctorate more than 40 years ago, the amino-acid sequence of one’s protein was not necessarily known and special collaborations were made to determine the sequence. This is generally now all done via gene sequencing not protein sequencing. Post-translational modifications can occur and in one’s electron density maps the missing electron density of amino-acid side chains can cause anxiety with respect to interpretation; debates such as on CCP4bb show the community view split between letting the *B* factors (atomic displacement parameters) inflate accordingly or truncating the side chain at that place where density is no longer visible. A complementary approach would be to measure the mass of the pure, final protein before crystallization. Indeed, mass spectrometry has grown impressively in its power and scope these last decades (see *e.g.* Liko *et al.*, 2016 ▸).

A milestone example of finding out the damage caused by excessive X-ray dose to acquire crystallography diffraction data was the work of Yano *et al.* (2005 ▸): X-ray absorption spectroscopy was used with a much lower dose to study reduction by X-ray-generated electrons of the manganese ions in the oxygen evolving complex of photosystem II (PSII) (Yano *et al.*, 2005 ▸). Specific X-ray damage to protein disulfides was shown much earlier, following a suggestion by Greg Petsko, by Helliwell (1988 ▸) and coworkers. This is now an extensive field of enquiry as measured for instance by the succession of radiation damage conferences, published in issues of the *Journal of Synchrotron Radiation*.

Infrared spectroscopy offers a variety of possible measurements to provide accuracy based on a precise crystal structure (see *e.g.* Barth, 2007 ▸). It can be applied to both static and time-resolved studies. These latter include taking the difference spectra between two structural states. As with colour changes, which feature pivotally in two of my case studies below, such difference spectra can be very informative and can confirm and complement what is seen with 3D structural probes.

In Section 5.6 I describe the use of solution small-angle X-ray scattering (SAXS) as a complement to cryocrystallography with a double aspect, confirming that the solution and solid-state crystal structures of a colouration protein are the same and, as well, that the cryo and room-temperature structures are the same. SAXS, and its neutron equivalent SANS, have very wide-ranging applications and are extensively described in recent books (Svergun *et al.*, 2013 ▸; Lattman *et al.*, 2018 ▸).

The connection between the molecular level of detail and the cellular level is bridged by the microscopies. The thickness of individual cells is problematic for electrons due to their being absorbed but not for X-rays. This yields a spatial resolution beyond the capability of light microscopy. A comparison of the capabilities of electron and X-ray microscopies is described by Du & Jacobsen (2018 ▸).

To end this section I choose an example from before protein crystallography had been brought to fruition by Max Perutz and John Kendrew. This is the case of sickle cell anaemia and haemoglobin (Pauling *et al.*, 1949 ▸). In the opening of their discussion Pauling *et al.* (1949 ▸) stated ‘On the nature of the difference between sickle cell anaemia hemoglobin and normal hemoglobin: Having found that the electrophoretic mobilities of sickle cell anaemia hemoglobin and normal hemoglobin differ, we are left with the considerable problem of locating the cause of the difference. It is impossible to ascribe the difference to dissimilarities in the particle weights or shapes of the two hemoglobins in solution: a purely frictional effect would cause one species to move more slowly than the other throughout the entire pH range and would not produce a shift in the isoelectric point. Moreover, preliminary velocity ultracentrifuge and free diffusion measurements indicate that the two hemoglobins have the same sedimentation and diffusion constants. The most plausible hypothesis is that there is a difference in the number or kind of ionizable groups in the two hemoglobins.’ Such changes were later shown by Max Perutz to have occurred and created sticky patches causing two haemoglobin molecules to hold together, distorting an erythrocytic cell and impeding blood flow. This is explained as a PDB-101 (Protein Data Bank, https://pdb101.rcsb.org/motm/41; see the section entitled Troubled Haemoglobins). This example shows the importance of 3D structure as well as the measurements made by Pauling *et al.* (1949 ▸).

## 3. Criticisms of the reductionist approach   

Overall there is scepticism of what we ‘atomic level structuralists’ do for biology. Dame Ottoline Leyser, Professor of Plant Development at the University of Cambridge, was quoted recently (Turney, 2019 ▸): ‘The defining feature of biology during the past few decades has been figuring out details of the parts. But biological systems don’t think they have parts’. Scepticism of the role of reductionism in understanding biology is a theme in Ernst Mayr’s book *What Makes Biology Unique*? (Mayr, 2007 ▸). Mayr even argues against the relevance of the discovery of the DNA double helix to understanding biology. As a counterpoint, a founding father of quantum mechanics, the physicist Erwin Schrödinger, posed the question ‘What is life?’ in his influential book *What is Life?: the Physical Aspect of the Living Cell*, in effect applying the physical sciences to this central question of biology (Schrödinger, 1943 ▸).

Then we have Sir Paul Nurse, awarded the Nobel Prize in Physiology or Medicine in 2001 for his work on the cell cycle of fission yeast [interview by Ireland (2014 ▸)]: ‘I work on the model organism fission yeast as I have done for 40 years. I’m taking synthetic and systems biology approaches to global cellular controls – that is, looking at the whole cell, particularly the cell cycle and cell shape, which are my areas of interest. Rather than get bogged down in detailed molecular descriptions of everything, I’m asking bigger questions like ‘how does a cell know how big it is?’, which has always fascinated me.’

So, where do I think these strong views stand today against our reductionist research in the molecular sciences within which crystallography is a key player, as are the microscopies and spectroscopies? More to the point, how can the crystallographer respond constructively, maybe only partly, to the concerns of the holistic biologists?

The issue of relevance starts when considering the protein crystalline state or the solution state of a protein but imagined placed inside the biological cell. NMR provides atomically detailed results in solution and of course protein crystallography provides atomic details for a protein in the solid state. The protein crystal is a curious hybrid of solid state, being an ordered lattice, with a large percentage of the crystal volume in solution, namely the solvent channels that run through the crystal.

Studies of the structure and function of an enzyme in the crystalline state were to my mind greatly facilitated by the invention of the flow cell (Wyckoff *et al.*, 1967 ▸). I describe a case study of the application of the flow cell in Section 5.2. This is a powerful approach to the issue of the relevance of solid-state results. It answers with a resounding yes the question of whether these crystal structure results are relevant to enzyme function. Flow cell results overcome then the objections of the NMR solution-state spectroscopists to the crystallographer’s results in the protein crystal.

Weaknesses in the armoury of crystallography remain such as crystallization conditions, to a greater or lesser degree, taking one’s results away from biological functioning conditions. As Lin (2018 ▸) recently stated: ‘Structure based drug design requires accurate structural information with the native conformation of a protein. However, scientists frequently select the protein that is suitable for crystallization but far from the physiological condition. It has been well known that protein conformation may be sensitive to the crystallization solution, and the different crystallization conditions or buffer selection can be a source of irreproducibility. It is a big challenge to find crystallization conditions that are close to the physiological conditions of a protein. To guide drug design with the native conformation of a protein, the developments of the proprietary crystallization screen kits with soft [‘soft’ in this context means to have a relatively non-perturbing impact on the protein structure] features for the protein to maintain its native conformation for drug target protein crystallization and new methods that can help select the best protein sample for crystallization trials are required.’

This caveat of Lin (2018 ▸) is perhaps more relevant than the one about having a lattice for a crystal. Lattice contacts in macromolecular crystals are rather few, whereas other possible effects of non-physiological crystallization conditions cannot be similarly dismissed as they affect all of the atoms in the crystallized protein.

A similar concern is the question about the strict relevance to biology of crystallography results now predominantly based on X-ray diffraction data measured at cryo temperature (Halle, 2004 ▸); see Section 4.1 below. This has been compounded by observations of specific X-ray damage to the crystallized protein. Conducting crystallography at physiologically relevant temperatures has then become an objective.

Neutron macromolecular crystallography (nMX), whilst pursuing protein structures with protonation states experimentally determinable, has also automatically yielded room-temperature structures. Given also that projects succeed in getting beamtime only where all other methods have failed, X-ray-, electron- or NMR-based, it is clear that in structural biology there is a strategic importance of this method. nMX has seen a sustained growth of the instruments, the software and methods at leading neutron sources (*e.g.* see Niimura & Podjarny, 2011 ▸; Cuypers *et al.*, 2013 ▸; Blakeley & Podjarny, 2018 ▸; Hoogerheide *et al.*, 2020 ▸), maximization of the potential of perdeuteration (Haertlein *et al.*, 2016 ▸), and application to resolving problematic protein structure–function studies (Helliwell, 2021 ▸).

The new femtosecond-range X-ray lasers yield X-ray diffraction data at room temperature and before the onset of radiation damage, the ‘diffract before the sample is destroyed’ approach (Neutze *et al.*, 2000 ▸). Synchrotron facilities are now also adopting the X-ray laser methods for delivery of streams of micron-sized samples and thereby also yielding results at physiological temperatures, albeit not free of radiation damage like the X-ray lasers. The use of streams of micron-sized crystals may be susceptible to variability within samples of the biological molecules being studied.

So, the physical methods of crystallography, microscopy and spectroscopy continue to strive for, and do clearly deliver, functionally relevant structural results. While many biologists have embraced molecular structure shall we ever convince holistic biologists? Our studies rarely take us directly to the whole organism. In Section 5.6 I give a case study where that all-important bridge to whole animals is achieved, the colouration of the lobster shell.

## 4. Structural artefacts   

### 4.1. Cryo versus room temperature   

Halle (2004 ▸) provided a landmark paper from the physical chemists’ point of view: ‘In biomolecular cryocrystallography: structural changes occur during flash-cooling: conformational switching of solvent-exposed side chains and weak ligand binding are likely artefacts…. Also the sites for molecular recognition, ligand binding or chemical catalysis, usually involve the solvent interfacial region where consequent cryo-artefacts are expected to be most pronounced.’ My own laboratory’s 1997 study (Deacon *et al.*, 1997 ▸) was cited by Halle (2004 ▸) where we had noted in our Section 3.9 that several amino-acid side chains (of the concanavalin A protein) had adopted largely different conformations; most of the changes were in poorly determined residues in the room-temperature structure, especially in the flexible loop regions; the number of detected solvent sites had more than doubled, to 319 at 110 K as compared with 149 at 293 K; and in particular there were 20 non-matching waters in the room-temperature structure which were connected to the movement of side chains by freezing out of the low-energy conformations of Asp82, Ser117, His121 (in a loop region), Lys135 and Thr196. But there were no major differences in either the protein or solvent structure around the saccharide-binding sites. Vigilance is needed for such artefacts. A distinction should of course be made between structural details that change (at the two temperatures) and things that become visible at the cryo temperature which were not visible before.

Is there then a science of what sort of structural artefacts can occur in proteins? In a recent review Fischer (2021 ▸) discusses practical aspects of preparing, acquiring and analysing X-ray crystallography data at room temperature. There is one, especially interesting, pioneering series of crystal structures of ribonuclease studied at nine temperatures (PDB codes 1rat through to 9rat) from 98 to 320 K (Tilton *et al.*, 1992 ▸). These were undertaken to scrutinize protein structure differences with temperature. Unfortunately the bound waters are not in these coordinates’ files and the structure factors likewise are unavailable. This illustrates the nature of the field of macromolecular crystallography which is still developing, *i.e.* crystal structures undertaken at 37°C should become more usual. Furthermore, can thermophilic proteins and extremophilic proteins be studied at these organisms’ temperatures, *i.e.* respectively, between 60 and 80°C and >80°C? Radiation damage by X-rays will presumably become the limiting factor but which can be circumvented by the use of neutrons or by NMR. As to feasibility, within an undergraduate physics project at York University in the mid-1980s, we showed that X-ray diffraction data could be recorded from a crystal of phenol insulin (Derewenda *et al.*, 1989 ▸) up to 50°C; at about 55°C the diffraction pattern disappeared but returned on cooling.

### 4.2. Bond distance and bond angle artefacts   

Electron crystallography is a developing method with increasing modern applications and scope in chemical (Gemmi *et al.*, 2019 ▸) and biological crystallography (see *e.g.* Clabbers & Abrahams, 2018 ▸; Gemmi *et al.*, 2019 ▸). But as Vainshtein (1964 ▸) pointed out it is unreliable when a crystal becomes too large, even at 1 µm, giving false distances and angles due to the multiple scattering of the electrons. In the modern era the study of Palatinus *et al.* (2015 ▸) illustrates the point well: working with a Ni_2_S nanocrystal of 0.1 µm they compared kinematical and dynamical treatments of the data analysis. The dynamical treatment was essential whereby: ‘The maximum distance to the corresponding atomic position in the reference structure decreased from 0.042 Å for kinematical refinement to 0.020 Å for dynamical refinement’. The application domain of electron crystallography will have to be carefully monitored according to sample size.

In protein crystallography a hidden artefact is inappropriate numbers of decimal places on non-covalent interatomic distances being presented in the figures of publications and where no standard uncertainty has been given on those distances. This absence of a formalism to calculate a standard uncertainty presumably led to the problem in the first place. Cruickshank (1999 ▸) started addressing this when he introduced the overall diffraction precision index (DPI) [see equation (1) below] which is widely applicable to studies made across a range of diffraction resolutions. The overall DPI for a study can be extended to individual atoms via equation (2) (Gurusaran *et al.*, 2014 ▸). What chance is there of reaching precision, let alone accuracy, if the error on the positions of a protein’s atoms is rarely considered?

Cruickshank’s form of the DPI is shown in (1), where σ(*x*, *B*_avg_) is the DPI for an atom with an average *B* factor, *N_i_* is the number of fully occupied atoms of type *i*, *p* = (*n*_obs_ − *n*_params_) is the number of observations minus the number of parameters for the atoms in the model, *C* is the completeness of the diffraction data, *R* is the *R* factor (or *R*_free_), *d*_min_ is the diffraction resolution and *k* is ∼1.0:
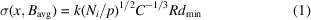


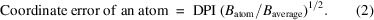



For low-resolution structures, the number of parameters may exceed the number of diffraction data. Then *p* = *n*_obs_ − *n*_params_ is negative, so that σ(*x*) is imaginary. Cruickshank (1999 ▸) circumvented this difficulty empirically by replacing *p* with *n*_obs_ and *R* with *R*_free_.

Since these papers were published there does seem to have been the good practice of non-covalent distances in the drawings of biological publications being at least quoted to an appropriate number of decimal places, usually guessed at previously, presumably.

### 4.3. Sample artefacts/variations   

At the International Symposium for Diffraction Structural Biology 2019 in Osaka, Joachim Frank, recipient of the Nobel Prize in Chemistry 2017, in his plenary lecture described the advantage of cryoEM is that, unlike a crystal, the molecular complex is in its natural state. His objection is to the crystal which involves the regular packing of those molecules, thereby trapping single states when there might be multiple states (Chen *et al.*, 2015 ▸). CryoEM offers a big advantage in having the capability to discern multiple states. This also seemed to me subject to the comment ‘true but’, *i.e.* trading being free of a crystal lattice for possible cryo-artefacts, which as yet is a topic incompletely understood. In any case, protein crystallographers like myself, during the course of research studies, will have encountered the situation where from one crystal to another of the same protein the diffraction data do not agree well enough to merge them together. This has been contained as a problem by using the dendrogram classification approach (Foadi *et al.*, 2013 ▸) but it also gives insights, by analysing different crystal clusters of a dendrogram, into the structural variations possible [*e.g.* see Fig. 6 of Foadi *et al.* (2013 ▸)]. The cryoEM approach works where multi-macromolecular complexes of a large size do not crystallize and it is a very powerful method of ‘seeing atoms’ as well as resolving multiple states.

### 4.4. Challenges to structure precision   

In both chemical and protein crystallography post-publication peer review shows that vigilance is required before a database entry can be trusted. Through the efforts of different groups of crystallographers in post-publication peer review of articles with their database downloaded files it has become apparent that there are numerous examples where corrections to crystal structures have been needed. These have been both at the level of improving the precision of deposited structures and of correcting inaccuracies on asserted structure–function relationships; see *e.g.* Rupp *et al.* (2016 ▸) for biological crystal structures. But it has not been the rule in biological crystallography for journal referees (and editors) to scrutinize articles with data with a PDB validation report. So it is perhaps unsurprising that database entries contain errors. Efforts have been made to improve the situation by at least describing the data science skills needed for biological X-ray crystallography referees (Helliwell, 2018 ▸). One benefit of such scrutiny is to point out to the editor that authors’ results often have unexplained density, which can then remain unmodelled and often uncommented on in a publication. The various aspects that can challenge structure precision described above can be further circumvented if the underpinning raw data are available (Helliwell *et al.*, 2017 ▸).

The divergence of the PDB from the Cambridge Structural Database (CSD) has also led to concerns by the CCDC (Cambridge Crystallographic Data Centre) itself about the quality of ligand structures in the PDB (Liebeschuetz *et al.*, 2012 ▸): ‘The good, the bad and the twisted: a survey of ligand geometry in protein crystal structures’. This has been reiterated by Jaskolski *et al.* (2021 ▸) in a study of nearly 100 coronavirus main protease crystal structures deposited in the PDB, who concluded with a list of problems associated with reliability of the deposited structures, and offered a list of procedural weaknesses of these database depositions.

The situation in chemical crystallography is much better because at least for IUCr journals in this area refereeing of articles with data with a *checkcif* report is done. In spite of this leadership for rigour by the IUCr, this is not always followed amongst other chemical journals. As Schwalbe (2018 ▸) remarked for high-resolution chemical crystallography: ‘Problems can arise in crystallographic databases with errors and omissions in the representation of data that impede searches, and with errors in the actual data. While the Cambridge Crystallographic Data Centre with its Improvement Projects has solved many of the first category of problems, errors in atomic coordinates and other crystallographic data are surprisingly common. Although modern software warns of many types of error, such errors appear even in recently deposited Crystallographic Information Files. Richard Marsh found many examples of missed symmetry in assignment of the space group; such errors are now waning. Hydrogen atoms are commonly placed in calculated positions. Particularly for OH and NH groups involved in hydrogen bonds, occupancy factors may need to be reduced to 0.5 or the hydrogen atom positions may require amendment. (For) imidazole derivatives sometimes only the consideration of bond distances and angles at the heteroatom can distinguish between OH or NH and unprotonated O or N. (There are cases of) mis-positioned hydrogen atoms in di­hydrogen phosphates and water aggregates as well as mis-identified elements.’

Expanding on this, Clegg (2021 ▸) describes a lack of reliability of chemical crystal structures, namely ‘examples of poor experiments, misinterpretation of data, scientific bias and preconceived ideas, incompetence and even deliberate fraud’.

So, before one can even consider crystal structure precision of a physical probe, let alone benchmarks towards accuracy, the reliability as measured by an absence of calculational errors is needed. This lack of reliability across biological and chemical crystal structure analysis also suggests that crystal structure teaching schools are needed more than ever and more widely.

Within the physics of diffraction, authors of a study generally focus on their ‘static’ model coordinates’ file derived from the processed ‘Bragg reflection intensities’ via their model refinement. The diffuse scattering outside the Bragg spots is generally not only uninterpreted but also uncommented on in article narratives. Where this is true these are missed opportunities to connect the structures and their dynamics. For overviews, and descriptions of the benefits of analysing the diffuse scattering, see Wall *et al.* (2018 ▸), Meisburger *et al.* (2020 ▸), and for a detailed example see de Klijn *et al.* (2019 ▸).

### 4.5. Prediction-of-structures artefacts or a more precise check on our molecular structural reality?   

The success of DeepMind in CASP14 [for a summary see Helliwell (2020 ▸)] raises interesting possibilities, pro and con. It predicts a protein’s fold based on its amino-acid sequence remarkably well. So, does DeepMind’s prediction mean that the deep learning’s collective wisdom of all the experimental protein crystal structures in the PDB at the time of CASP14, *i.e.* its learning set of structures, achieves the collective precision of all of those? Does this collective precision then reach close to accuracy, as I have defined it in my introduction? More detail is awaited on how DeepMind’s algorithms work. But from the DeepMind blog https://deepmind.com/blog/article/AlphaFold-Using-AI-for-scientific-discovery the public at large can learn that DeepMind’s methods ‘relied on deep neural networks that are trained to predict properties of the protein from its genetic sequence. The properties our networks predict are: (*a*) the distances between pairs of amino acids and (*b*) the angles between chemical bonds that connect those amino acids. The first development is an advance on commonly used techniques that estimate whether pairs of amino acids are near each other.’

The success of DeepMind in CASP14 stirred me to reflect on our foundations of protein crystallography (Helliwell, 2020 ▸); I have written: ‘it is the detailed protein structure that determines function, not the protein fold *per se*, but DeepMind are clearly entering the area of positioning atoms in detail but not really with a clear level of precision at the atom by atom detailed level. Secondly, multi-domain proteins are currently outside of this achievement. Thirdly, where a structure is known, I note that structural dynamics studies start, *i.e.*, in effect, while there may be one fold, there isn’t one structure in function terms! In our experimental arena, kinetic crystallography is set to thrive with an expansion of X-ray lasers, the ESRF EBS (Extremely Brilliant Source), Diamond II *etc.* building on many earlier developments. In addition diffuse scattering is all there for the measuring and interpreting, *i.e.* structural dynamics again. Onto more specific topics: a third of all proteins are metalloproteins. Predicting where a metal might bind and which metal element it is, is not solved. And not just metal ions but all sorts of other ligands too will still have to be done experimentally.’

It was gratifying how DeepMind carefully acknowledged the experimentally determined structures of the protein crystallographers. A worldwide effort had gone into the synchrotron X-ray sources in the 1980s and 1990s and onwards to develop the multiple-wavelength method (Okaya & Pepinsky, 1956 ▸) for protein crystal structure phase determination. A resume of that work at the SRS (Synchrotron Radiation Source) Daresbury, ESRF BM14, Elettra and at CHESS, amongst others, was given by Cassetta *et al.* (1999 ▸) and also the general phasing idea involving seleno­methio­nine (Hendrickson *et al.*, 1990 ▸) has been incredibly valuable.

## 5. Case studies   

### 5.1. Element identification   

The two case studies in this section show that the precision of a crystal structure can be challenged not only in the standard uncertainties of the bond distances and angles but also in being sure of the identity of metal atoms. In protein crystallography in particular, synchrotron radiation can allow a fairly rapid and effective check of the content of a new protein crystal on the beamline using the X-ray fluorescence spectrum. This is well applied at the ESRF for example (Leonard *et al.*, 2009 ▸). Without this it was shown in a retrospective, *i.e.* post-publication, that reanalyses of the metal sites were necessary in a large proportion of cases and which led to improvements in existing PDB depositions (Grime *et al.*, 2020 ▸). This study used ion beam analysis through particle-induced X-ray emission (PIXE), which quantitatively identified the metal atoms in 30 previously structurally characterized proteins. Over half of these metals had been misidentified in the deposited structural models and by using the correct metal their structural models were improved.

#### 5.1.1. The location of manganese and calcium ion cofactors in pea lectin crystals by use of anomalous dispersion and tunable synchrotron X-radiation   

From the outset of harnessing synchrotron radiation in crystallography the tunability was exploited to identify which metal atom was which. The study by Einspahr *et al.* (1985 ▸) used the enhancement of the anomalous dispersion of the manganese ions with a wavelength near the Mn *K* absorption edge on the tunable, focused X-ray spectrometer for protein crystallography at the Daresbury Synchrotron Radiation Source. Accurate identification and location of the Mn^2+^ and Ca^2+^ ions was possible, based on the large relative difference in *f* ′′ anomalous components of the ions in the anomalous difference Fourier maps; such was not the case, based on the relative difference in atomic numbers of the ions, in the native electron density map.

#### 5.1.2. Determination of zinc incorporation in the Zn-substituted gallophosphate ZnULM-5 by multiple-wavelength anomalous dispersion techniques at 11 X-ray wavelengths   

**Figure 3:**
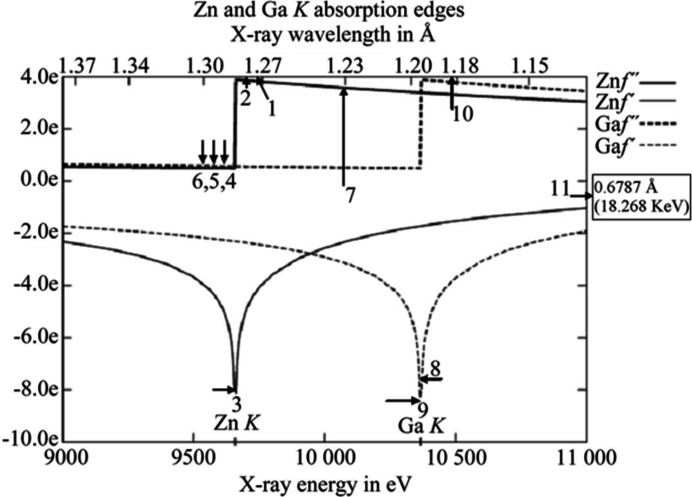
Determination of zinc incorporation in the Zn-substituted gallophosphate ZnULM-5 by multiple-wavelength anomalous dispersion techniques. Absorption edges of zinc and gallium, with X-ray wavelength positions of the 11 single-crystal X-ray diffraction data sets indicated by the arrow labels. These two curves show the real and imaginary parts of the X-ray scattering from an atom that vary with wavelength for each element (Helliwell *et al.*, 2010 ▸).

The most X-ray wavelengths in an element identification study to my knowledge is that of Helliwell *et al.* (2010 ▸). The location of isomorphously substituted zinc over eight crystallographically different gallium sites was determined in a single-crystal study of the gallophosphate ZnULM5 involving the measurement of single-crystal diffraction data sets around the *K* edges of both Ga and Zn, as well as two reference data sets away from each absorption edge (see Fig. 3 ▸). With these it was possible to selectively exploit dispersive differences of each metal atom type in turn, which allowed the major sites of Zn incorporation to be identified. As the crystal was non-centrosymmetric, with space group *P*2_1_2_1_2, it was also possible to use anomalous differences to corroborate the results obtained from the dispersive differences. These results were obtained firstly from difference Fourier maps, calculated using a phase set from the refined structure from data measured at the Zr *K* edge. Also, refined dispersive and anomalous occupancies, on an absolute scale, could be obtained using the program *MLPHARE* (Collaborative Computational Project, Number 4, 1994 ▸), allowing estimates for the Zn incorporation of approximately 22 and 18 at.% at the *M*1 and *M*3 sites, respectively, to be obtained. *JANA2006* (Petricek *et al.*, 2014 ▸) also allowed the ready determination of standard uncertainties on the occupancy parameters, which for *M*1 and *M*3 were 20.6 (3) and 17.2 (3) at.%, respectively.

### 5.2. Realizing ultimate precision via transfer of ligand charge density results to protein as ligand binder   

Whilst a charge density study of a protein crystal structure, especially of functional or medicinal importance, is generally not possible, a charge density crystal structure study of the ligand on its own may be viable. Furthermore, its electrostatic charge density distribution can be considered as a direct description of the protein’s binding site if it is known that the ligand binds well in a competitive assay with the protein’s natural substrate. A pioneering example of this kind is that of Malińska *et al.* (2014 ▸) of sunitinib malate, an inhibitor of tyrosine kinases and approved as a drug in 2006. As the authors remark: ‘To obtain a deeper understanding of the interactions that are present in molecular complexes, *i.e.* beyond geometrical considerations following standard crystal structure determination, analysis of charge-density distribution is desirable.’ This approach has considerable potential to indirectly extend the precision of a protein crystal structure to an ultimate level.

### 5.3. Enzyme catalysis in the crystal: hy­droxy­methyl­bilane synthase (Helliwell *et al.*, 1998 ▸)   

**Figure 4:**
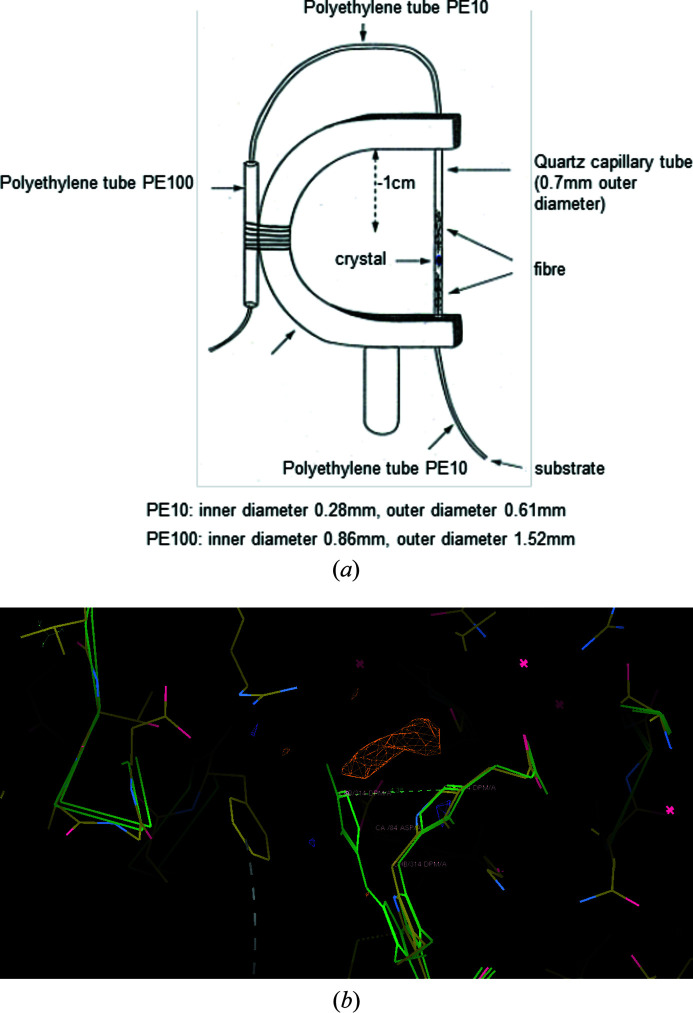
Time-resolved enzyme crystallography. (*a*) Diagram of flow cell with a yoke support based on Wyckoff *et al.* (1967 ▸) for time-resolved enzyme crystallography [Nieh (1997 ▸) based on Petsko (1985 ▸)]. (*b*) The coordinates and structure factors of the 2 h model are 1ypn and the reduced cofactor HMBS (1ah5) showing that the time-resolved 2 h structure cofactor (1ypn) is also in the active state. The time-resolved 2 h electron density, contoured at 3σ (shown in orange), is in the active site adjacent to and above the side chain of Asp84, which plays a pivotal role throughout the catalytic reaction cycle. Also superimposed is the oxidized cofactor HMBS (PDB code 1pda), which is an inactive form of the enzyme. This figure was made with *Coot* (Emsley & Cowtan, 2004 ▸).

Utilizing the flow cell concept of Wyckoff *et al.* (1967 ▸) the structure of the catalytically active, reduced form of the enzyme hy­droxy­methyl­bilane synthase (HMBS, Lys59Gln mutant) was studied by Laue dffraction at the ESRF as the substrate, porphobilinogen (PBG), was fed to an immobilized crystal in a flow cell of our design [Fig. 4 ▸(*a*)]. Laue diffraction data were measured at a variety of time points up to 4 h. For another time point, with the substrate supply to a crystal in the flow cell having been stopped at 4 h, monochromatic data were collected at 12 h 30 min on ESRF BM14 (*i.e.* about 8 h after the substrate supply was stopped). The difference Fourier electron density maps showed that extended electron density appeared in the active-site region of the di­pyrrole cofactor. Fig. 4 ▸(*b*) shows the reduced cofactor [PDB codes 2ypn (Nieh *et al.*, 1999 ▸) and 1ah5 (Hädener *et al.*, 1999 ▸)], proximal to the oxidized C2 ring position (PDB code 1pda; Louie *et al.*, 1992 ▸). Most significantly this electron density was adjacent to and above the side chain of Asp84, which is known to play a pivotal role throughout the catalytic reaction cycle. That the electron density was not visible in the 12 h case showed that the product of the enzyme-catalysed reaction had been released into the crystal solution channel. By the time of the conclusion of this experiment, after 12 h, the crystals had changed from colourless to red. This was a significant, complementary observation to the electron density map changes in the enzyme active site. Subsequent to these experiments the human HMBS enzyme has been studied by static X-ray crystallography in the apo and ES2 forms by trapping of this state (Pluta *et al.*, 2018 ▸; Kallio *et al.*, 2021 ▸; Sato *et al.*, 2021 ▸). The two approaches, time-resolved and static crystallography, yield complementary views of the intermediate structures, ES2. The static, trapped intermediate, crystal structures yield the most detail. The use of site-directed mutants of course does risk structural artefacts compared with the native enzyme structure. Overall this is an incredibly interesting enzyme with its complex mechanism fundamental to life. Namely, the addition of different metals to the cyclic ring forms haem, chloro­phylls and vitamin B12. In humans, deficiency in the activity of this enzyme is directly associated with the dominant hereditary disease acute intermittent porphyria, more commonly known as the ‘madness of King George’. This is another direct link from the molecular level to a holistic biology, in this case a medical condition.

### 5.4. The predictive force of precise crystal structures – an iodo­platin for tumour therapy (Tanley & Helliwell, 2014 ▸)   

That crystal structure studies have predictive force is an implicit and explicit statement of exploring structure and function relationships. In trying to connect with the whole living animal the following example involving anti-tumour therapy is a good one. In an extensive theme of platin binding to proteins we unexpectedly came across an iodoplatin. This arose because of the chemical conversion of cisplatin and carboplatin cocrystallized with a model protein (hen egg white lysozyme) crystallized under sodium iodide conditions. The theme involved over 30 different crystal structures. It showed that the platins chemically bound through the platinum atom to a protein’s amino acids (notably histidine) besides their nucleic acid structural chemistry, the former being side effects and the latter the actual anti-cancer therapy. One of our structures in particular, an iodo­platin bound to the protein, showed how a dual photon energy approach targeting the iodine and the platinum *K* edges would allow one to vary the penetration depth of X-rays into a tumour pre-loaded with the iodoplatin. Cisplatin administration has previously been studied in this way involving radiation therapy using a synchrotron source and the Pt *K* edge alone (Biston *et al.*, 2004 ▸; see also https://www.esrf.fr/UsersAndScience/Publications/Highlights/2004/Imaging/Ima10). To undertake such a dual *K*-edge radiation therapy involving iodinated cisplatin or carboplatin under patient conditions would build upon those experiments.

### 5.5. The use of protein powder diffraction and crystallography for the characterization of insulin polymorphs and analogues   

The characterization of pharmaceutical samples which are absorbed by a patient in bulk form, such as insulin crystalline slurries, can only be done by powder diffraction. Karavassili *et al.* (2017 ▸), focusing on X-ray protein powder diffraction, reviewed research findings on human insulin microcrystals exhibiting polymorphism upon physicochemical modifications of their environment (*i.e.* pH, ligand binding). Four new biologically active types of human insulin crystals were identified, and their structures successfully determined by a combination of powder and single-crystal diffraction measurements. Such research continues for pharmaceutical products containing microcrystals with improved activity and stability for patient benefit.

On the single-crystal side, seeking to improve a medical treatment, the high-resolution crystal structure of a fast-acting human insulin analogue, glulisine, has been determined (Gillis *et al.*, 2021 ▸). The key molecular level comparisons between this crystal structure of glulisine and of previous insulin crystal structures showed that a unique position of the glutamic acid, not present in other fast-acting analogues, pointed inwards rather than to the outside surface. This reduces interactions with neighbouring molecules and so increases preference of the more-active-for-patients dimer form, giving then a better understanding of the behaviour of glulisine. An unexpected finding was that the glulisine formulation is documented as a zinc-free insulin analogue for its rapid absorption action. Insulin crystallography has shown that zinc is pivotal for hexamer formation. The new glulisine crystal structure showed zinc bound in the same way as in native insulin, by three histidine amino acids. This finding must mean that traces of zinc ions are present in the commercial, as-supplied, formulation solution. A further optimization for glulisine is now clear, that of finally removing the zinc.

The full scope of protein powder diffraction, including to larger proteins, is described by Margiolaki (2019 ▸).

### 5.6. Determining the molecular mechanism of the colouration of a live animal, the lobster   

In this effort to rise to the challenges laid down by the holistic biologists, Ottoline Leyser, Ernst Mayr and Paul Nurse, I describe the molecular mechanism of the colouration of a live animal, the study of the lobster shell being a particularly valid connection between the molecular level and an animal.

**Figure 5:**
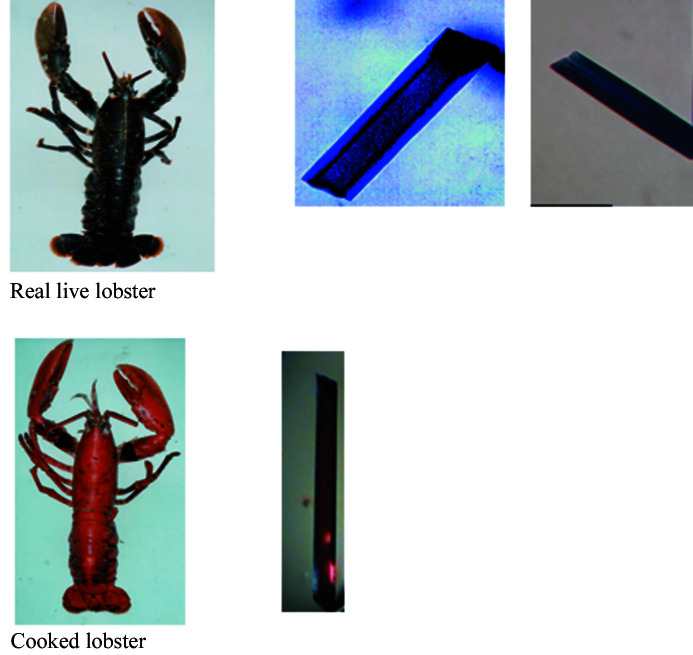
Comparison of the colours of a live lobster with crystals of crustacyanin as well as a cooked lobster and a crystal of astaxanthin. Top row: live lobster, β-crustacyanin (Chayen, 1998 ▸) and α-crustacyanin crystals (Chayen *et al.*, 2003 ▸). Bottom row: cooked lobster and an astaxanthin crystal grown by Dr M. Helliwell (Bartalucci *et al.*, 2007 ▸).

Spanning more than 50 years the methods of biochemistry, biological crystallography, spectroscopy, solution X-ray scattering and microscopy have been applied to study the molecular basis of the colouration of the live lobster. The blue colouration of the carapace of the European and American lobsters is provided by a multi-molecular carotenoprotein, α-crustacyanin. The European lobster (*Homarus gammarus*) crustacyanin has been the most extensively studied. Its α-crustacyanin complex extracted from the lobster carapace is a 16-mer of different protein subunits, each binding the carotenoid, astaxanthin (AXT). The biological purpose of the coloured shell is unknown although it may be a means of camouflage against predators such as octopus. A breakthrough in the structural studies came from the determination of the crystal structure of β-crustacyanin comprising the protein subunits A1 with A3 with two shared bound astaxanthins (Cianci *et al.*, 2002 ▸). This crystal structure was based on diffraction data measured at 100 K. The crystal was blue at room temperature and blue at cryo temperature from which we can conclude that the 3D structure of the β-crustacyanin is free of cryo-artefacts, at least in terms of the protein astaxanthin molecular interactions. Furthermore solution X-ray scattering (SAXS) measurements of the β-crustacyanin showed an excellent agreement with the predicted SAXS curve from the cryo crystal structure (Chayen *et al.*, 2003 ▸). So, we can conclude that the crystalline state and the solution state structures of β-crustacyanin agree. The importance of colour as a simple but effective assay for a real-life connection is illustrated in Fig. 5 ▸ whereby the mechanism of change in colouration on cooking a lobster is explicitly shown and can be compared directly with the colour of a pure astaxanthin crystal, which matches the cooked lobster, and the live lobster whose colour matches the colours of the crustacyanin crystals.

## 6. Technological innovations matching the modern needs   

Research and development of technology directions are highly exciting for structural molecular sciences researchers these days. These technology developments are truly extensive and include: new ultra-brilliant SR (synchrotron radiation) sources known as ‘ultimate storage rings’ (MAX IV, PETRA III, NSLS II, the ESRF Extremely Bright Source, with other upgrade plans for ALBA, Diamond, PETRA IV and the Swiss Light Source for example); the X-ray lasers [Linac Coherent Light Source (LCLS), FERMI, SACLA] are into their second generation with the LCLSII and the EuroXFEL; the new neutron sources JPARC, SNS and ESS as well as the ILL’s upgrade programmes; new electron microscopy and electron crystallography capabilities; new higher-field NMR machines (1.2 GHz). These developments are accompanied by continued highly impressive detector developments such as those by the commercial companies.

The case studies I have described in this article as examples from the fields of structural biology and chemistry are being built upon with experiments with these new technologies. The use of the flow cell approach of Wyckoff *et al.* (1967 ▸) is now reaching a much wider range of projects because crystal samples can now be as small as 1 µm (Schmidt, 2013 ▸). This means the diffusion time for the reactants to initiate the catalysis by an enzyme is orders of magnitude improved. This makes it feasible to use faster enzymes than hitherto for time-resolved crystallography. There is also a wide adoption of the serial crystallography approach at the new synchrotron beamlines, not only for time-resolved crystallography, but also for room-temperature crystal structure determination.

The development of neutron sources and instruments is not only speeding up throughput of projects, but also widening their scope via larger unit-cell capabilities for neutron macromolecular crystallography, and thereby the molecular-weight ceiling is raised. CryoEM is also being enhanced by widening its scope to smaller molecular-weight systems. One can expect an overlap of capabilities of cryoEM with neutron studies as well as with X-rays.

## 7. Conclusions   

I have brought to a focus in this article the views of several biologists who are sceptical of the molecular level approach. By quoting them I hope I have shown that their objections can be replied to constructively, if not exactly rebutted, by the crystallographer. Forming a constructive response is as much a state of mind and philosophy, and some might declare it unnecessary. But I think it is important, as important as explaining to the public and school children what we do. With this outlook and with our careful choice of projects, both societally relevant as well as curiosity driven, the investment in our field represented by our technological resources can continue to improve.

By adopting an overview of the probes of the structure of matter that we deploy I hope that I have also shown just how synergistic the one is with the other. Each probe however has at least one overriding advantage. X-ray crystal structure analysis offers direct ways to solve the structure in the first place by overcoming the phase problem. Neutrons yield structures complete with hydrogens, and for biological crystallography this means with deuteriums. Electrons offer the chance to work with ultra-small samples, down to the single molecule. There are then ways to cross-check the crystalline state structural results are meaningful by solution scattering or time-resolved crystallography. NMR can be applied to determine the extent of disorder in a crystal as well as for structure and dynamics determination in solution. CryoEM yields precise structures for multi-macromolecular complexes and their multiple states. Arising from ‘small-molecule’ chemical crystallography I have described the exciting changes deriving from the charge density structures as being transferrable, and indeed with direct relevance to molecular recognition of a ligand binding to a biological structure. The expansion of capability of materials powder diffraction into the protein powder diffraction domain is I think also a remarkable development. An unexpected new resource is the prospect of protein structure prediction of the DeepMind kind, which we are eager to learn more about.

When coupled together, the precision of the methods used in structural science achieves accuracy. Unlike in physics training though, there is a middle ground between precision and accuracy where we can achieve predictive force from a precise structure which is in effect a form of accuracy. In similar vein the reductionist approach reaches towards the whole organism and in its physiological state.

All these aspects open new doors to change and extend the foundations of the structural molecular sciences.

## Figures and Tables

**Table 1:** Fundamental comparison of X-rays and electrons

X-rays	Electrons
Yields electron density	Yields electron potential map
Diffraction efficiency relatively low with respect to electrons	Diffraction efficiency high, good for very small samples, otherwise strongly absorbed and thereby:
Mature method, well understood and validated (but still needs article with data files and *checkcif* report for submission of chemical crystallography articles to IUCr journals†)	Prone to multiple scattering which can seriously affect bond distances and angles
	CryoEM and electron crystallography still improving their capabilities as methods
Phase problem	No phase problem‡
Radiation damage effects	Radiation damage effects

† That procedure is to be extended to IUCr’s biological journals as well in 2021.

‡ In protein electron crystallography the phase problem is discussed by Gemmi *et al.* (2019 ▸) and, as they summarize, molecular replacement is so far the method applied.

**Table 2:** Practical challenges for X-rays, electrons and neutrons

X-rays	Electrons	Neutrons
Radiation damage such as splitting of disulfides, truncation of amino-acid side chains, changes to oxidation states of metal atoms/ions	Very strong interaction with matter, an advantage for very thin/small samples such as a single molecule or a nanocrystal	Weak flux, so: (i) Use as broad a bandpass of the emitted neutrons as possible, *i.e.* Laue diffraction (ii) Use as long a mean wavelength as possible to increase scattering efficiency (iii) Reduce any background scattering so as to maximize signal to noise (*i.e.* in biology change hydrogens for deuteriums with their 40× less incoherent scattering) (iv) Grow as big a crystal as possible, *e.g.* ∼1 mm^3^ (typical range 0.1 to 8 mm^3^) (v) Maximize the full exploitation of perdeuteration
This leads to use of cryo temperature to (partially) mitigate these effects	Microcrystals have strong electron beam absorption/multiple scattering and any bigger samples cannot be used with an electron beam, in transmission at least	
	Careful scrutiny of the error estimates on bond lengths and angles needed	
